# Effect of repeat refresher courses on neonatal resuscitation skill decay: an experimental comparative study of in-person and video-based simulation training

**DOI:** 10.1186/s41077-023-00244-5

**Published:** 2023-02-25

**Authors:** Julia M. McCaw, Sarah E. Gardner Yelton, Sean A. Tackett, Rainier M. L. L. Rapal, Arianne N. Gamalinda, Amelia Arellano-Reyles, Genevieve D. Tupas, Ces Derecho, Fides Ababon, Jill Edwardson, Nicole A. Shilkofki

**Affiliations:** 1 Department of Anesthesiology and Critical Care Medicine, Johns Hopkins University, Baltimore, MD USA; 2 Department of Medicine, Johns Hopkins University, Baltimore, MD USA; 3 Department of Pediatrics, Southern Philippines Medical Center, Davao City, Philippines; 4 Operation Smile Philippines Foundation, Inc.—Mindanao Cleft Center, Davao City, Philippines; 5 Department of Anesthesiology, Makati Medical Center, Makati, Philippines; 6 Department of Pediatrics, College of Medicine, Davao Medical School Foundation Inc., Davao City, Philippines; 7 Department of Obstetrics and Gynecology, College of Medicine, Davao Medical School Foundation, Inc., Davao City, Philippines; 8 Department of Gynecology and Obstetrics, Johns Hopkins University, Baltimore, MD USA; 9 Department of Pediatrics, Johns Hopkins University, Baltimore, MD USA

## Abstract

Neonatal deaths are a major contributor to global under-5-year-old mortality. Training birth attendants can improve perinatal outcomes, but skills may fade over time. In this pilot study, we assessed skill decay of nursing students after remote video versus in-person resuscitation training in a low-resource setting. Filipino nursing students (*n* = 49) underwent traditional, in-person simulation-based Helping Babies Breathe (HBB) training in Mindanao, Philippines. Participants were then assigned to receive refresher training at 2-month intervals either in-person or via tele-simulation beginning at 2 months, 4 months, or 6 months after initial training. A knowledge examination and practical examination, also known as objective structured clinical examination B in the HBB curriculum, were administered before retraining to assess knowledge and skill retention at time of scheduled follow-up. Time to initiation of bag-mask ventilation (BMV) in seconds during simulated birth asphyxia was the primary outcome. Skill decay was evident at first follow-up, with average time to BMV increasing from 56.9 (range 15–87) s at initial post-training to 83.8 (range 32–128) s at 2 months and 90.2 (range 51–180) s at 4 months. At second follow-up of the 2-month group, students showed improved pre-training time to BMV (average 70.4; range 46–97 s). No statistical difference was observed between in-person and video-trained students in time to BMV. Because of COVID-19 restrictions, the 6-month follow-up was not completed. We conclude that remote video refresher training is a reasonable alternative to traditional in-person HBB training. Our study also suggests that refreshers may be needed more frequently than every 2 months to mitigate skill decay. Additional studies are necessary to assess the longitudinal impact of tele-simulation on clinical outcomes.

**Supplementary Information:**

The online version contains supplementary material available at 10.1186/s41077-023-00244-5.

## Background

Worldwide, neonatal deaths make up an increasing percentage of the under-5 mortality rate. Training birth attendants in low- and middle-income countries (LMICs) is known to be a cost-effective strategy to improve neonatal mortality rates [1]. Helping Babies Breathe (HBB) has been shown to reduce early neonatal mortality by as much as 47% in LMICs through a hands-on simulation curriculum that stresses the importance of initiating lifesaving interventions within 60 s after delivery, referred to as the “Golden Minute” [2–5]. Although initial program evaluation has shown reassuring mortality trends secondary to HBB training, there is growing concern that resuscitation skills, such as time to initiate bag-mask ventilation (BMV), decay over time [5–8]. Some studies have reported improvements in pass rates for standardized practical examination, known as objective structured clinical examination B in the HBB curriculum, after refresher training or daily supervised practice [9–11]. Many births in LMICs, particularly those with community-based traditional birth attendants (TBAs), occur outside of the hospital and thus outside of the physical and organizational infrastructure necessary to support frequent traditional, in-person trainings. Geographic distance has also been described as a barrier to frequent simulation necessary to minimize decay in neonatal resuscitation skills in the USA [12]. Currently, the ideal frequency of refresher training to ensure competence in essential neonatal resuscitation skills is not well-described for hospital-based providers or community-based TBAs.

With nearly 20% of births worldwide still occurring in the absence of skilled health personnel, [13] continued training of birth attendants is crucial. However, the maintenance of resuscitation skills by TBAs is limited not only by infrequent exposure to birth asphyxia but also geographic boundaries adding to travel costs and separation of TBAs from local duties, including patient care [6, 14]. One approach to overcome these multiple constraints is through tele-simulation using synchronous video communication that connects trainers and trainees. A recent pilot study of hybrid in-person and telehealth HBB training in Guatemala not only showed similar post-training practical examination pass rates but also was well received by learners and saved approximately US $3979 [15], suggesting that tele-simulation may be a feasible and cost-conscious option for LMICs.

The Philippines is an LMIC with a persistently high neonatal mortality rate of 13 deaths per 1000 live births [16]. The country is also one of the most digitally connected LMICs, with 57% of all Filipinos owning smartphones [17] and 43% using the Internet [18] in 2019. These metrics suggest that Internet-based interventions could be introduced and accepted relatively widely. The relationship of one investigator (NS) with multiple HBB master trainers in the Philippines facilitated the implementation of training sessions with local support, including support from the Mati City Health Office in the Mindanao region of the Philippines. With investments in Internet connectivity and planned infrastructure under development to allow community health stations, the ability to participate in a centralized tele-psychiatry program based in Mati, the potential exists for additional Internet-dependent health interventions to reach TBAs in the low-resource areas where they live and work, including HBB training.

Before implementing fully remote HBB training, it was necessary to both uncover any potentially deleterious effects on skill or knowledge decay related to tele-simulation and attempt to define the ideal frequency of refresher training for maintenance of HBB knowledge and skills. In partnership with Davao Oriental State College of Science and Technology (DOSCST; Mindanao, Philippines), we trained nursing students in HBB and planned refresher trainings at set intervals. Our aim was to compare skill decay of nursing students who received scheduled in-person HBB refresher training to that of nursing students who received refresher training delivered via synchronous video coaching. The time to initiate BMV after birth was the primary outcome. Secondary outcomes included knowledge score on the written HBB multiple choice examination and overall practical examination score.

## Methods

Second-year nursing students at DOSCST (*n* = 49) were trained in HBB by a collaborative group of both US-based and local HBB master trainers using the 2nd edition HBB curriculum (Laerdal, 2017) and low-fidelity Laerdal NeoNatalie manikins (Laerdal, Wappingers Falls, NY, USA). This initial training was carried out in person in November 2019. We chose second-year nursing students as they were preparing to start their clinical rotations and would be entering the delivery room where their skills could be measured in real-life scenarios post-training. Study participants signed an informed consent statement in accordance with Johns Hopkins University School of Medicine Institutional Review Board (IRB no. 00104685) with reciprocal approval by Davao Oriental State University administration and ethics committee (no formal IRB process in place at time of study).

**Fig. 1:**
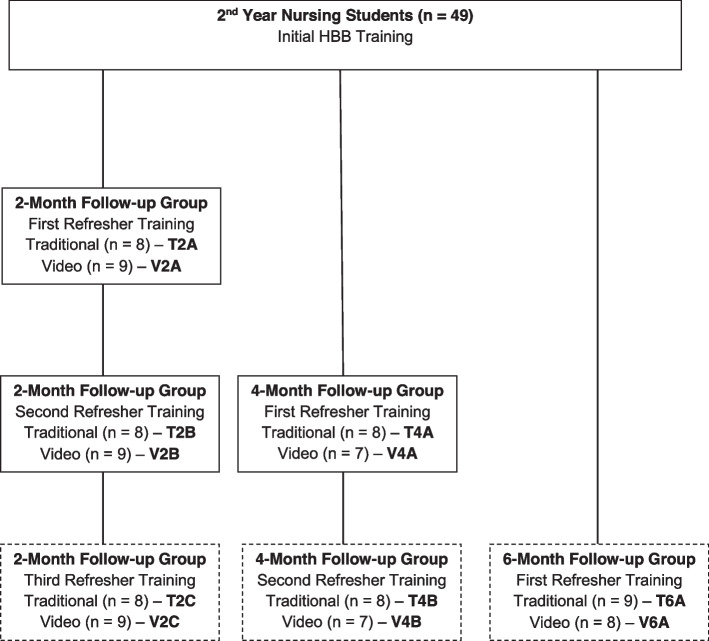
Participant group assignment and follow-up frequency. Schematic of participant group assignments and study design after initial in-person training. HBB, Helping Babies Breathe. Dashed outlines indicate that the refresher training was cancelled because of COVID-19 restrictions

After initial training, nursing students were randomized into 2-month, 4-month, and 6-month follow-up groups and then further divided within these time-based groups into remote video (V) or traditional in-person (T) follow-up testing and training (Fig. 1). These assignments resulted in a total of six novel groups: T2 (*n* = 8), V2 (*n* = 9), T4 (*n* = 8), V4 (*n* = 7), T6 (*n* = 9), and V6 (*n* = 8). T2 and V2 would return for follow-up and refresher training at 2 months, 4 months, and 6 months, T4 and V4 would return at 4 months and 6 months, and T6 and V6 would return at 6 months only. The first follow-up was designated by “A,” second follow-up by “B,” and third follow-up by “C.” All groups would return at 1 year for final testing of knowledge and skill decay.

Simulation training and testing for the initial course was held in the DOSCST nursing school. The pre-training and post-training evaluations, which included a paper-based, multiple-choice HBB knowledge examination [19] and modified practical examination (Additional file 1), were administered individually for each student. The practical examination was modified for our study to include exact time in seconds to initiation of BMV to allow identification of skill decay at a more granular level compared to the binary (yes/no) assessment of time to BMV traditionally used in HBB training. Pre-training scores on the HBB knowledge examination were collected just before initial training. Each trainer documented the individual pre-training practical examination score of each student by hand, using a digital stopwatch to record time to initiate BMV after simulated delivery, defined by trainer stating “baby born.” After participants completed the full HBB training curriculum, which consists of interactive lectures and hands-on simulator practice, we documented initial post-training HBB knowledge score, practical examination score, and time to BMV for each student. The in-person trainer gave each student individual feedback regarding technique and adherence to HBB protocol immediately after the initial practical examination and had the option to demonstrate proper technique. Students were allowed to repeat the practical examination an infinite number of times to ensure skill proficiency, but only the first score post-training was considered when reporting study results. If a student did not pass the practical examination on their first attempt, they were required to repeat the examination until they achieved a passing score. Students did not have access to simulators between sessions to minimize confounding factors of interim practice for study purposes.

**Fig. 2:**
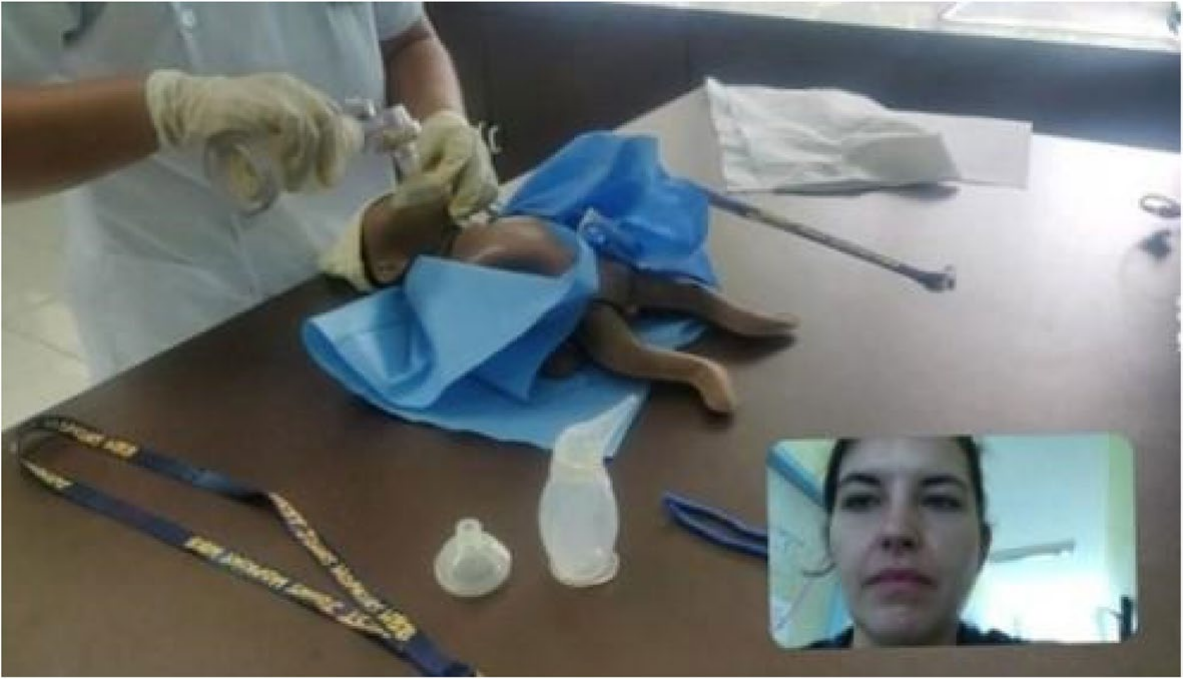
Screenshot of tele-simulation showing participant and instructor during practical examination

We tested video clarity and Internet connection for video calls during the training of master trainers 3 months before study implementation. At that time, Facebook Messenger video chat was found to be the most reliable video connection between DOSCST in the Philippines and Baltimore, MD, USA. This platform was agreed upon with local trainers to be the preferred method of video communication during future remote trainings. During the 2- and 4-month follow-ups, DOSCST students connected with trainers via video on a laptop computer with webcam (Logitech HD Webcam C270) using local Internet connection. The trainer was alone in another classroom on the DOSCST campus with a personal iPhone 6 (Apple Corp., Cupertino, CA, USA) connected via local Internet (Fig. 2).

Relative proximity of student and trainee was planned to allow for troubleshooting of video and Internet connection, if necessary, with plans for extension to Baltimore-Mindanao connection for future video trainings. During video trainings, the remote trainer used an additional NeoNatalie simulator and bag-valve-mask ventilator to demonstrate skills and technique adjustments over video.

At scheduled refresher trainings, the HBB knowledge examination and practical examination were administered as in initial training, enabling assessment of retention. Refresher trainings were brief, lasting approximately 5–10 min; following initial practical examination assessment, one-on-one coaching and demonstration of BMV skills were provided as needed either in-person or over video, depending on assigned study group. Post-training practical examination score and time to BMV were collected to ensure appropriate skill uptake after refresher training. Data was analyzed using Stata software (StataCorp, College Station, TX, USA). Baseline differences between groups were calculated by ANOVA, differences in continuous outcomes were calculated by two-sample *t*-test, and differences in proportions were calculated by Fisher’s exact analysis.

## Results

### Initial training

Initial differences in outcomes between study groups and improvement after training are shown in Table 1. No significant differences were detected between groups for baseline mean time to initiate ventilation, knowledge examination scores, or practical examination scores. Of note, before HBB training, only four students were able to successfully perform BMV, but no students were able to perform BMV within 60 s after simulated birth of the infant.

**Table 1:** Comparison of skills and knowledge of Filipino nurses before in-person HBB training

Parameter	Pre-training	*p*-value for difference between groups, pre-training*
Time to initiate BMV, seconds, mean (SD)	103 (10)	0.89 (*n* = 4)
Successful BMV, *n* (%)	0 (0)	NA
Knowledge examination score, mean (SD)	12.5 (2.1)	0.95
Knowledge examination pass rate, *n* (%)	20 (40.8)	0.62
Practical examination score, mean (SD)	3.29 (1.8)	0.53
Practical examination pass rate, *n* (%)	0 (0)	NA

* HBB* Helping Babies Breathe, *BMV* Bag-mask ventilation, *NA* Not applicable, *SD* Standard deviation

*Difference between groups: analysis of variance; knowledge examination pass rate: Fisher's exact test. Proportions of successful BMV and practical examination pass rates were unable to be calculated given a denominator of zero. Of note, at initial assessment, only 4 participants were able to appropraitely ventilatre, but all well past the acceptable 60s mark

 For reference, a score of “pass” for the practical examination requires appropriately completing 13 out of 18 tasks, including completion of all four essential tasks (see Additional file 1), and a passing score on the HBB knowledge examination requires at least 14 of 18 questions answered correctly. See additional files for copy of the practical examination.

Immediately after training, 33 (67.3%) students were able to successfully perform BMV within 60 s on first attempt, with an average time of 56.9 (range 15–87) s. Six (12.2%) score sheets post-training documented time to BMV as “ < 60 s” or “ > 60 s” in line with the traditional assessment of the HBB practical examination (rather than our modification), limiting inclusion of this data in our analysis of exact time to BMV. We accounted for these data points using “successful BMV” proportions to compare those students achieving BMV at < 60 s to those who achieved it at ≥ 60 s. Average knowledge scores and practical examination scores also improved after training, with 47 (95.9%) and 46 (93.8%) students, respectively, passing these tests.

### Refresher trainings

COVID-19 restrictions on gatherings at DOSCST and local/international travel bans prevented us from completing the 6- and 12-month follow-up sessions as planned. As a result, we collected follow-up data for only four novel groups (T2, V2, T4, and V4). Students demonstrated a significant improvement in time to initiate BMV after each training session (Table 2).

**Table 2:** Effect of training on time to bag-mask ventilation in 2-month study group (T2 + V2) at each training session

	Time to bag-mask ventilation (seconds)^a^		
Parameter	Pre-training	Post-training	*p*-value†
Initial training	104.0 (12.2)	56.2 (13.0)	< 0.001
First refresher training	83.8 (29.8)	46.1 (10.1)	< 0.001
Second refresher training	70.4 (13.1)	48.7 (8.6)	< 0.001

^a^Data are presented as mean (SD)

†Data analyzed by *t*-test

Comparisons of primary and secondary outcomes for groups followed up at 2 months and 4 months are shown in Table 3. The data in this table illustrate knowledge retention after the initial training, before either in-person or video refresher training, which was introduced at these sessions. At the 2-month follow-up (T2A and V2A; Fig. 1), knowledge examination scores were maintained; however, skill decay was notable, with only 4 (23.5%) students able to perform BMV successfully within 60 s with a group average of 83.8 (range 32–128) s. This decline translated to a lower practical examination pass rate (76.5%), which takes into consideration whether the participant completed essential tasks within the scored practical examination (see Additional file 1). At the first follow-up for the 4-month group (T4A and V4A; Fig. 1), similar trends were noted. Students achieved a 100% pass rate on the knowledge examination but exhibited further skill decay. Three students (20%) achieved successful BMV in under 60 s. However, with the average time at 90.2 (range 51–180) s, practical examination pass rates dropped to 53.3% before refresher training. This pass rate was significantly lower than the pre-refresher practical examination pass rate of 2-month participants (*p* = 0.02). There was no statistically significant difference at first follow-up between 2-month and 4-month groups in time to ventilation (*p* = 0.11), knowledge score (*p* = 0.95), or practical examination score (*p* = 0.14).

**Table 3:** HBB refresher training outcomes: differences between 2-month and 4-month follow-up groups

Parameter	2-month follow-up (T2 + V2)		4-month follow-up (T4 + V4)		*p*-value
	Post-initial training	Pre-refresher training	Post-initial training	Pre-refresher training	
Time to initiate BMV, seconds, mean (SD)	56.2 (13)	83.8 (29.8)	49.0 (15.7)	90.2 (35.4)	0.11
Successful BMV, *n* (%)	11 (64.7)	4 (23.5)	11 (73.3)	3 (20.0)	0.76
Knowledge examination score, mean (SD)	16.4 (1.2)	16.6 (1.2)	16.2 (1.3)	16.4 (0.9)	0.95
Knowledge examination pass rate, *n* (%)	17 (100)	16 (94.1)	15 (100)	15 (100)	0.10
Practical examination score, mean (SD)	15.9 (1.5)	14.8 (1.4)	16.8 (1.3)	14.7 (1.8)	0.14
Practical examination pass rate, *n* (%)	14 (82.3)	13 (76.5)	14 (93.3)	8 (53.3)	0.09

*HBB* Helping Babies Breathe, *BMV* Bag-mask ventilation

Differences between groups were calculated by subtracting values during pre-training at follow-up from initial post-training values (in Table 1) and applying *t*-test or Fisher’s exact test. For description of groups, please see Fig. 1. For reference, a score of “pass” for the practical examination requires appropriately completing 13 out of 18 tasks, including completion of all four essential tasks (see Additional file 1), and a passing score on the HBB knowledge examination requires at least 14 of 18 questions answered correctly. Of note, four participants (26.7%) who initially passed pre-training declined testing post-training.

### Decay

Skill decay was evident as early as 2 months, as previously described. Even at the start of the second refresher course for the 2-month groups (T2B and V2B), average practical examination score had declined from 16.3 (*SD* 0.8) at first refresher to 14.8 (*SD* 2.0). Time to initiate BMV improved from 83.8 (range 32–128) s at the start of the first refresher training session to 70.4 (range 46–97) s at the start of the second refresher training session (*p* = 0.05). Average HBB knowledge examination score also improved from 16.6 (*SD* 1.2) at the first refresher to 17.6 (*SD* 0.6) at the second refresher.

Table 4 shows a comparison of decay between the traditional-(T) and video-trained (V) groups. We compared the change in student performance from T2A post-training to T2B pretraining with the corresponding change from V2A to V2B. We found no statistically significant difference between the traditionally trained and video-trained groups in time to ventilation (*p* = 0.536), knowledge examination score (*p* = 0.523), or practical examination score (*p* = 0.273).

**Table 4:** Average skill and knowledge decay of traditional (T, in-person) and video (V, remote) groups

Outcome	*T* decay	*V* decay	*p*-value*
Time to BMV, seconds, mean (SD)	27.7 (16.1)	20.4 (24.2)	0.54
Knowledge examination score, mean (SD)	1.3 (1.8)	0.8 (1.1)	0.52
Practical examination score, mean (SD)	− 2.3 (2.6)	− 1.0 (1.8)	0.27

Decay was measured by subtracting post-training results at first refresher follow-up from pre-training results at second follow-up

*BMV* Bag-mask ventilation, *SD* Standard deviation

*Calculated by *t*-test

## Discussion

Previous studies suggest that frequent HBB refresher trainings may be necessary for retention of resuscitation skills [3, 20]. However, for many providers, gathering for frequent training is limited by travel time/distance and associated costs. In this study, we compared skill decay between Filipino nursing students who trained in person and those who trained via tele-simulation over remote video. We found no significant difference in decay of knowledge or skills between these two groups, suggesting that video refresher training is a potential alternative to traditional in-person instruction for future HBB trainings.

By 2 months after the initial training, students did not retain the ability to successfully perform BMV by 60 s after delivery, with average time to BMV increasing from 56.2 (*SD* 13) to 83.8 (*SD* 29.8) s. At the subsequent follow-up, held 2 months after the first refresher training, knowledge scores had improved, but overall practical examination score had declined, suggesting further skill decay. However, when we took a more granular look at skill decay, average time to BMV pre-training had improved by 13.4 s from the first refresher course (average 83.8, range 32–128 s) to the second refresher course (average 70.4; range 46–97 s). This finding suggests that repeat refresher training could alleviate skill decay for this essential step of resuscitation, and that a more granular examination of resuscitation skills essential for actual delivery room practices may be a more pragmatic way to assess skill decay. Indeed, this was our hope in choosing time to BMV as our primary outcome in this study.

Our observation of retained knowledge but initial skill decay with improvement after repeat refresher training is in line with other recent studies supporting multiple refreshers. A recent systematic review [5] and studies of resuscitation skills among healthcare providers in the US [21, 22] have defined multiple refresher trainings (i.e., low-dose high frequency) as a reasonable approach to ensuring skill retention. Demonstrating the ability to obtain similar results in low-resource settings is important because use of tele-simulation may allow for more frequent refresher trainings for TBAs limited by travel distance and therefore increased long-term skill retention. With appropriate resources (Internet connection, additional manikins, and bag-valve-mask ventilators), tele-simulation could also be a feasible alternative in future pandemics during “stay-at-home” orders that limit travel and close contact that are necessary for traditional simulation-based training in geographically isolated areas.

Another approach apart from consecutive refreshers that could be considered is earlier initiation of refresher follow-up training. One of the initial HBB follow-up studies by Bang et al. showed that practitioners returning for retesting at 1–5 months after initial training had less risk of failing practical skill examinations than those returning at 7–9 months (*OR* = 0.5) [7]. To our knowledge, no studies on HBB have explicitly examined refresher timing earlier than 2 months. Nevertheless, this frequency may be necessary given the decay already evident at this time point in our study. A 2020 study by Oermann et al. that assessed adult CPR skills of nursing students daily, weekly, monthly, and quarterly showed that those who had daily or weekly training acquired compression and ventilation skills more quickly [23]. The retention of skills after four consecutive sessions was not assessed, making extrapolation of skill decay beyond a month difficult. Other available studies to date on the topic of resuscitation that have examined effects of refresher training conducted earlier than 2 months post-training have also included a more frequent training schedule, which makes it difficult to interpret whether such frequent training is necessary for retention of skills or if earlier refresher introduction with less frequent training may be helpful. Additional studies are necessary to establish the ideal frequency for refresher training and to assess the feasibility of more frequent training and potential associated costs in low-resource settings.

Apart from limitations on full study completion due to travel and gathering restrictions during the COVID-19 pandemic, other factors may limit application of our findings to more general populations. For unspecified reasons, four participants who passed the practical examination during pre-training testing declined retesting post-training at the 4-month follow-up. This discrepancy did not affect decay data but would have limited our ability to calculate decay of 28.6% of students at the 6-month follow-up. This discrepancy in trainer assessment starting at the 4-month follow-up may suggest that even master trainers may need refreshers on proper training techniques and data collection. The need for such training should be considered in future studies. Another potential limitation affecting generalizability of our results was our choice to study nursing students and perform testing at their place of study to prevent significant attrition, which has hampered previous studies of midwives and other community-based birth attendants [5, 9]. We are hopeful that the growing cyberstructure in Mindanao can be utilized for future studies to test remote video training of community health workers and encourage retention of participants. Furthermore, this study introduced video instruction at refresher trainings but utilized in-person instruction at initial training. Given the lack of experience of our trainees, establishing proper BMV skills in person was essential to development of foundational skills. A hybrid approach, as described by Jones-Bamman et al. [15], may be a reasonable alternative; additional studies that examine initial training over video are necessary to assess feasibility of fully remote training. Additionally, we recognize that video training is dependent on reliable synchronous video communication, which may be a barrier to adoption in many remote settings. Future studies should explore the role of asynchronous video assessment and instruction, which is less dependent on timely and consistent Internet connections, but could affect efficacy of debriefing and its impact on knowledge and skill retention. Other limitations of this study include language barriers, which were mitigated by participation of local trainers and widespread use of English as the modality for medical instruction, and the competing occupational responsibilities of trainers that affected their availability to serve as instructors. Future studies are necessary to assess the longitudinal impact of neonatal resuscitation skills taught via tele-simulation on actual delivery room use of skills, neonatal mortality rates, and other clinical outcomes.

## Conclusions

Remote video refresher training using tele-simulation is a potential alternative to traditional in-person HBB training. Resuscitation training may be needed more frequently than every 2 months to mitigate skill decay. Additional studies are necessary to assess the longitudinal impact of tele-simulation on clinical outcomes.

## Supplementary Information

**Additional file 1. **“Modified Practical Examination” with instructions, scoring guide, and essential steps needed to obtain passing score. Note modification from traditional HBB Objective Structured Clinical Examination B with addition of “**PLEASE RECORD TIME OF START” in skill description and “At___seconds” under trainer’s documentation of skill performance.

**Additional file 2. **“Data Summary by Participant” Knowledge = Knowledge Examination; GM = golden minute; Practical= Practical Examination; NA = “not applicable”, used in the case of participant not completing the task or lacking documentation. Assigned “Group” as described in manuscript, designated by either Traditional “T” or Video “V” and scheduled initial follow up designated by number (2-month, 4-month, 6-month). “A” denotes first follow-up, completed by T2, V2, T4, V4 groups; “B” denotes second follow-up, completed by T2, V2 groups.

## Data Availability

The dataset supporting the conclusions of this article is included within the additional files attached to this article.

## Abbreviations

BMV: Bag-mask ventilation
COVID-19: Coronavirus disease 2019
CPR: Cardiopulmonary resuscitation
DOSCST: Davao Oriental State College of Science and Technology
HBB: Helping Babies Breathe
LMIC: Low- and middle-income countries
SD: Standard deviation
TBA: Traditional birth attendant
USD: United States dollars
