# Correction: Effect of repeat refresher courses on neonatal resuscitation skill decay: an experimental comparative study of in-person and video-based simulation training

**DOI:** 10.1186/s41077-023-00252-5

**Published:** 2023-04-17

**Authors:** Julia M. McCaw, Sarah E. Gardner Yelton, Sean A. Tackett, Rainier M. L. L. Rapal, Arianne N. Gamalinda, Amelia Arellano-Reyles, Genevieve D. Tupas, Ces Derecho, Fides Ababon, Jill Edwardson, Nicole A. Shilkofski

**Affiliations:** 1 Department of Anesthesiology and Critical Care Medicine, Johns Hopkins Univer, sity, Baltimore, MD USA; 2 Department of Medicine, Johns Hopkins Un, iversity, Baltimore, MD USA; 3 Department of Pediatrics, Southern Philippines Medical Center, Davao City, Philippines; 4 Operation Smile Philippines Foundation, Inc.—Mindanao Cleft Center, Davao City, Philippines; 5 Department of Anesthesiology, Makati Medical Center, Makati, Philippines; 6 Department of Pediatrics, College of Medicine, Davao Medical School Foundation Inc, Davao City, Philippines; 7 Department of Obstetrics and Gynecology, College of Medicine, Davao Medical School Foundation, Inc, Davao City, Philippines; 8 Department of Gynecology and Obstetrics, Johns Hopkins University, Baltimore, MD USA; 9 Department of Pediatrics, Johns Hopkins University, Baltimore, MD USA

**Correction: Adv Simul 8, 7 (2023)**

**https://doi.org/10.1186/s41077-023-00244-5**

The original article [1] contained a typo in author, Nicole A. Shilkofski's name which has since been amended.

## References

[1] McCaw JM (2023). Effect of repeat refresher courses on neonatal resuscitation skill decay: an experimental comparative study of in-person and video-based simulation training. Adv Simul.

