# Supplementary material for: Effect of repeat refresher courses on neonatal resuscitation skill decay: an experimental comparative study of in-person and video-based simulation training
Source: Adv Simul (Lond). 2023 Feb 25;8:7. doi: 10.1186/s41077-023-00244-5 (PMC9959951; doi:10.1186/s41077-023-00244-5)
Supplement: Supplementary file 1 — Additional file 1. “Modified Practical Examination” with instructions, scoring guide, and essential steps needed to obtain passing score. Note modification from traditional HBB Objective Structured Clinical Examination B with addition of “**PLEASE RECORD TIME OF START” in skill description and “At___seconds” under trainer’s documentation of skill performance. [file 41077_2023_244_MOESM1_ESM.docx]

**Helping Babies Survive: Helping Babies Breathe**

**Modified Practical Examination: Bag and Mask Resuscitation**

| **Bag and Mask Resuscitation- Skill Check**  **Instructions for the Examiner**   - Briefly review the instructions for the participant - Have necessary equipment and supplies nearby - ****Note the time between birth and beginning ADEQUATE ventilation (i.e. chest rise is seen by examiner) - Observe only; do not intervene in demonstration of the participant - The feedback will be held at the end of the assessment for all participants   **Read the following to the learner:**  I will read a case- please listen carefully and then show me how you would care for the baby. I will indicate the baby’s response with the simulator or in words. I will provide no other feedback until the end of the case.  “You are called to assist at the birth of a 38 week gestation baby. You arrive two minutes prior to birth. Introduce yourself and show what you will do.” |
| --- |

Participant ID#__________________________ Date____________________

| **Checklist of Skills** | | | | Yes **Performed to standard** | No **Did NOT perform to standard** | |
| --- | --- | --- | --- | --- | --- | --- |
|  |  |  |  | Check appropriate box for each | | |
| 2.1 | Prepares the area for delivery, area for ventilation | | |  |  | |
|  | Checks Equipment is functional (BVM etc) | | |  |  | |
|  | Cleans hands and/or places gloves on | | |  |  | |
| Prompt: *“After 2 minutes give baby to learner and say, “The amniotic fluid is clear. Show how you will care for the baby.” ***START TIMER NOW* | | | | | | |
| 2.2 | | Dries baby thoroughly | |  |  | |
|  | | Removes wet cloth and replaces with dry cloth | |  |  | |
| **Prompt:** Show or say, *“The baby is not crying.” “You see some secretions in mouth”* | | | | | | |
| 2.3 | | Keeps baby warm during resuscitation (hat, cover) | |  |  | |
| 2.4 | | Positions head, **clears airway BY SUCTIONING CORRECTLY MOUTH FIRST THEN NARES** | |  |  | |
| 2.5 | | Stimulates breathing by rubbing the back | |  |  | |
| 2.6** | | Evaluates breathing **RECOGNIZES BABY IS NOT BREATHING** | |  |  | |
|  | | **SKILL** | | Yes **Performed to standard** | No **Did NOT perform to standard** | |
| 2.7 | | Cuts cord and moves to area for ventilation OR ventilates by mother with cord intact | |  |  | |
| 2.8 | | Starts ventilation within The Golden Minute (ADEQUATELY WITH GOOD CHEST RISE VISIBLE)  *****PLEASE RECORD TIME OF START** | | **At ________**  **seconds** | **At ______ seconds** | |
| 2.9** | | **VENTILATES AT 40 BREATHS/MINUTE** (30-50 acceptable) | |  |  | |
| 2.10** | | **LOOKS at exposed chest FOR CHEST MOVEMENT** | |  |  | |
| **Prompt:** *Show or say, “The baby is not breathing.”* | | | | | | |
| 2.11 | | | Evaluates breathing and recognizes baby is not breathing |  |  |  |
| 2.12 | | | Calls for help |  |  |  |
| 2.13 | | | Continues ventilation |  |  |  |
| Prompt: Say, *“Please show what to do if the chest is not moving with ventilation.”* (OR OBSTRUCT AIRWAY BY HOLDING VENTILATION BULB). After one or more steps to improve ventilation, say *“The chest is moving now” (OR RELEASE OBSTRUCTION).* | | | | | |  |
| 2.14** | | | **IMPROVES VENTILATION BY DOING ANY OF:**  Head- repositions head, reapplies mask  Mouth-clears secretions, opens mouth slightly  Bag-squeezes bag harder |  |  |  |
| **Prompt:** Show or say, “The baby is not breathing; heart rate is normal.” | | | | | |  |
| 2.15 | | | Evaluate breathing **and heart rate by feeling cord**  Recognizes baby not breathing but heart normal |  |  |  |
| 2.16 | | | Continues ventilation |  |  |  |
| **Prompt:** After 3 minutes say, *“The heart rate is 120 per minute and the baby is breathing.”* | | | | | |  |
| 2.17 | | | Recognizes baby is breathing and heart rate is normal |  |  |  |
| 2.18 | | | Stops ventilation; monitors baby and communicates with mother |  |  |  |

# Score ______/ 18 2.6. 2.9 2.10 2.14 all ticked? Yes / No Pass / Fail

# (circle one) (circle one)

# TRAINER INITIALS ___________
